# Modeling AeroForm tissue expander for postmastectomy radiation therapy

**DOI:** 10.1002/acm2.12682

**Published:** 2019-07-22

**Authors:** Elaine Dziemianowicz, Stephen J. Gardner, Karen Chin Snyder, Ning Wen, Eleanor M. Walker, Correen Fraser, Anne Reding, Indrin J. Chetty

**Affiliations:** ^1^ Department of Radiation Oncology Henry Ford Health System Detroit MI USA

**Keywords:** AAA, Acuros, AeroForm, dose calculation, high density, postmastectomy radiation therapy, tissue expander

## Abstract

The AeroForm chest wall tissue expander (TE) is a silicon shell containing a metallic CO_2_ reservoir, placed surgically after mastectomy. The patient uses a remote control to release compressed CO_2_ from the reservoir to inflate the expander. AeroForm poses challenges in a radiation therapy setting: The high density of the metallic reservoir causes imaging artifacts on the planning CT, which encumber structure definition and cause misrepresentation of density information, in turn affecting dose calculation. Additionally, convolution‐based dose calculation algorithms may not be well‐suited to calculate dose in and around high‐density materials. In this study, a model of the AeroForm TE was created in Eclipse treatment planning system (TPS). The TPS model was validated by comparing measured to calculated transmission through the AeroForm. Transmission was measured with various geometries using radiochromic film. Dose was calculated with both Varian’s Anisotropic Analytical Algorithm (AAA) and Acuros External Beam (AXB) algorithms. AAA and AXB were compared using dose profile and gamma analyses. While both algorithms modeled direct transmission well, AXB better modeled lateral scatter from the AeroForm TE. Clinical significance was evaluated using clinical data from four patients with AeroForm TEs. The AeroForm TPS model was applied, and RT plans were optimized using AAA, then re‐calculated with AXB. Structures of clinical significance were defined and dose volume histogram analysis was performed. Compared to AXB, AAA overestimates dose in the AeroForm device. Changes in clinically significant regions were patient‐ and plan‐specific. This study proposes a clinical procedure for modeling the AeroForm in a commercial TPS, and discusses the limitations of dose calculation in and around the device. An understanding of dose calculation accuracy in the vicinity of the AeroForm is critical for assessing individual plan quality, appropriateness of different planning techniques and dose calculation algorithms, and even the decision to use the AeroForm in a postmastectomy radiation therapy setting.

## 1 INTRODUCTION

For breast cancer patients with greater than four positive lymph nodes, positive or close margins, or a tumor greater than 5 cm, the National Comprehensive Cancer Network guidelines recommend postmastectomy radiation therapy (PMRT). Currently there is no consensus on the optimal method and timing of postmastectomy breast reconstruction for patients receiving PMRT.^1^, ^2^, ^3^ One common technique is the two‐stage breast reconstruction. In this method, a tissue expander (TE) is placed at the time of mastectomy. In the following weeks, the TE is gradually expanded to the desired size, and PMRT is delivered after expansion is complete. At a later time, the TE is exchanged for a permanent implant.^4^, ^5^, ^6^

Several types of TEs are available. The CPX® (Mentor, Irvine, CA, USA), Natrelle® (ALLERGAN, Santa Barbara, CA, USA), Dermaspan^TM^ and AlloX_2_® (Sientra, INC., Santa Barbara, CA, USA) TEs consists of a silicon shell containing a magnetic injection port. The TE is expanded by externally aligning a magnetic port locating device to the internal magnetic port (IMP). Once aligned, saline is injected percutaneously through the IMP to inflate the TE. Patients receive weekly injections, over the course of 6–8 weeks.^7^, ^8^

The AeroForm TE (AirXpanders, Palo Alto, CA, USA) consists of a silicon shell containing a stainless steel reservoir of compressed CO_2_. The TE is expanded using a hand‐held remote control, which sends a radio‐frequency signal to the expander. The TE in turn releases small volumes of compressed CO_2_ from the reservoir into the silicon shell. The expansion is patient controlled; typically a patient releases 10 cc CO_2_ at a time, up to three times per day, over the course of 4–6 weeks. If radiation is indicated, the patient is typically simulated and treated after expansion is complete.^8^, ^9^

Tissue expanders pose particular challenges in a radiation oncology setting. The high‐density metallic components of both types of TE cause artifacts in treatment planning CT images. The degradation of image quality makes target definition more challenging, and misrepresentation of CT number in and around the TE may cause errors in dose calculation. 16‐bit CT reconstruction corrected using metal artifact reduction (MAR) reconstruction techniques may minimize the effects of artifacts on image quality.^10^, ^11^ However, even with accurate CT information, convolution dose calculation algorithms are not designed for use with high‐Z materials.^12^, ^13^

Previous literature has proposed various solutions dealing with TEs in a radiation treatment planning systems (TPS).^14^ Chen *et al.* and Yoon *et al.* studied the dosimetric impact of TEs utilizing an IMP.^15^, ^16^ Both authors corrected the CT image by applying density overrides to the IMP and surrounding artifacts. They defined the IMP using known physical dimensions of the magnetic disk, and a density override determined by transmission measurements. Chen *et al.* found good agreement between transmitted depth‐dose profiles measured with film and dose calculated using the Anisotropic Analytical Algorithm (AAA) (Varian Medical Systems, Palo Alto, CA, USA).^15^ Yoon *et al.* described good agreement between transmitted depth‐dose profiles measured with TLDs and dose calculated using the Collapsed Cone Convolution (CCC) algorithm (Philips Healthcare, Fitchburg, WI, USA).^16^

Manufacturers of the AeroForm TE recommend defining the device in the TPS and applying appropriate density overrides.^9^ In a 2014 study, Moni *et al* evaluated the dosimetric impact of AeroForm TE in Varian Eclipse TPS (Varian Medical Systems, Palo Alto, CA, USA).^17^ Moni placed OSLDs at various locations around the AeroForm TE on anthropomorphic phantom. The set‐up was imaged and an RT plan was created and delivered. The accuracy of AAA with and without heterogeneity corrections was evaluated by comparing the calculated dose to the OSLD‐measured dose. Moni reported agreement between AAA and measured data within 5%, except at the reservoir‐chest wall‐interface, where the measured dose was consistently 5–15% higher than calculated. Moni was also unable to observe the predicted “dose shadow” effect with OSLD measurements, possibly due to uncertainty in detector placement.^17^ These areas are of significant clinical importance; clinicians require accurate dose calculation in these regions to assess overall RT plan quality.

In a 2014 study, Tran *et al* measured transmission through various components of the AeroForm CO_2_ reservoir using an ion chamber.^18^ The authors reported physical density assignments in Pinnacle TPS that resulted in dose calculations in agreement with measured transmission data. Significant differences existed between the experimentally determined density and vendor‐reported density. Using an anthropomorphic phantom with the AeroForm TE, Tran *et al* compared dose calculated in Pinnacle TPS to dose measured with TLDs at various locations. Percent differences between calculated and measured doses ranged between −10 and +15%.^18^

This study deals with the accuracy of modeling of the Aeroform TE in Eclipse TPS using both AAA and Acuros External Beam (AXB) algorithms (Varian Medical Systems, Palo Alto, CA, USA). AAA is under the general class of superposition/convolution algorithms in which total dose is computed by superposition/convolution of the primary dose with scatter dose kernels. Such algorithms account indirectly and approximately for electron transport in heterogeneous media. AAA uses a 3D pencil‐beam kernel, which accounts for changes in electron density perpendicular to the beam direction by applying radiological depth scaling.^19^ The AXB algorithm is considered a grid‐based Boltzmann Solver (GBBS) as it analytically solves the Boltzmann transport equation using interaction cross sections specific to the relevant material and energy. The electron fluence spectrum in each voxel is computed, and dose is subsequently determined by integration over energy of the product of the electron fluence spectrum and the relevant cross section within the voxel, divided by the mass density of the voxel.^20^ As such, the Acuros AXB characterizes electron transport, albeit approximately, more like a Monte Carlo‐based algorithm. A GBBS algorithm such as AXB may therefore better suited to calculate dose near the various heterogeneous boundaries of the AeroForm TE.

Convolution algorithms such as Pinnacle CCC and Eclipse AAA underestimate dose perturbation occurring in the presence of high‐density materials. The most significant inaccuracies occur near the boundaries of the high‐density object, where effects of electron backscatter and lateral scatter are not accounted for. AAA also underestimates attenuation from high‐density materials.^12^, ^13^

For patients with TE that uses an IMP, the known inaccuracies of convolution algorithms may have a limited clinical impact.^21^, ^22^ The high‐density magnet is generally more than 1 cm away from patient tissue, so inaccuracies in lateral scatter dose are confined to the saline filling the TE and the IMP itself. AAA’s underestimation of attenuation through the IMP might affect the accuracy of the dose calculated in the “dose shadow” region. However, as discussed previously, this effect can be mitigated by assigning an experimentally determined relative electron density (RED) value for the IMP in the clinician’s TPS.

The AeroForm TE employs a geometry where known inaccuracies of a convolution algorithm may have a greater clinical significance. The high‐density CO_2_ reservoir is only separated from the patient chest wall by the thin silicon shell of the TE. A convolution algorithm’s underestimation of lateral scatter in this region might lead to a hot spot in the chest wall adjacent to the reservoir that is not evident in the calculated dose distribution. As with the IMP TEs, a convolution algorithm might also underestimate the effect of the dose shadow. Measurements discussed previously by Moni *et al* and Tran *et al* are not inconsistent with these predictions. However, it is possible that a GBBS algorithm such as AXB, might provide more accurate dose calculations in these clinically significant areas. Studies have shown that AXB achieves accuracy similar to Monte Carlo, even in and around high‐density heterogeneities.^13^

This study aims first to describe a method for modelling the AeroForm in Eclipse TPS. It is the authors’ hope that the procedure can be easily implemented into any clinician’s radiation therapy TPS. The TPS model will be optimized and evaluated for both AAA and AXB, employing techniques described Chen et al and Yoon et al. Finally, the dosimetric impact of the AeroForm TE will be evaluated using clinical patient data. Differences in plan outcome based on dose calculation algorithm will be discussed. An understanding of dose calculation accuracy in the vicinity of the AeroForm TE is critical for assessing individual plan quality, appropriateness of different planning techniques and dose calculation algorithms, and even the decision to use the AeroForm TE in a PMRT setting.

## 2 MATERIALS AND METHODS

### 2.1 Imaging and modeling the AeroForm TE

**Figure 1:**
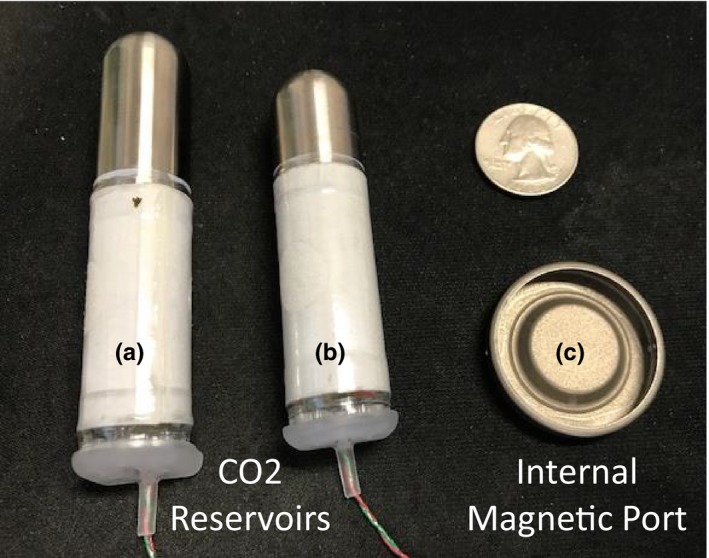
(a) CO_2_ reservoir for 800 cc AeroForm (b) CO_2_ reservoir for 400 and 600 cc AeroForm (c) Magnasite magnetic injection port.

The AeroForm TE is available in three sizes: 400, 600, and 800 cc. The 400 and 600 cc sizes utilize a CO_2_ reservoir 7.6 cm in length and 1.9 cm in diameter. The 800 cc TE uses a reservoir 9.0 cm in length and 1.9 cm in diameter. Both sizes of AeroForm reservoir are shown in Fig. 1, with the Natrelle® IMP for comparison.

Each reservoir size was imaged using a Philips Brilliance Big Bore CT scanner (Philips Health Care, Cleveland, OH, USA). The AeroForm reservoir was aligned such that the long axis was perpendicular to axial plane, so as to minimize artifacts. Helical CTs were acquired for a 20 cm FOV with a 1 mm slice thickness using 120 kVp and 500 mAs. A 16‐bit reconstruction was generated.

**Figure 2:**
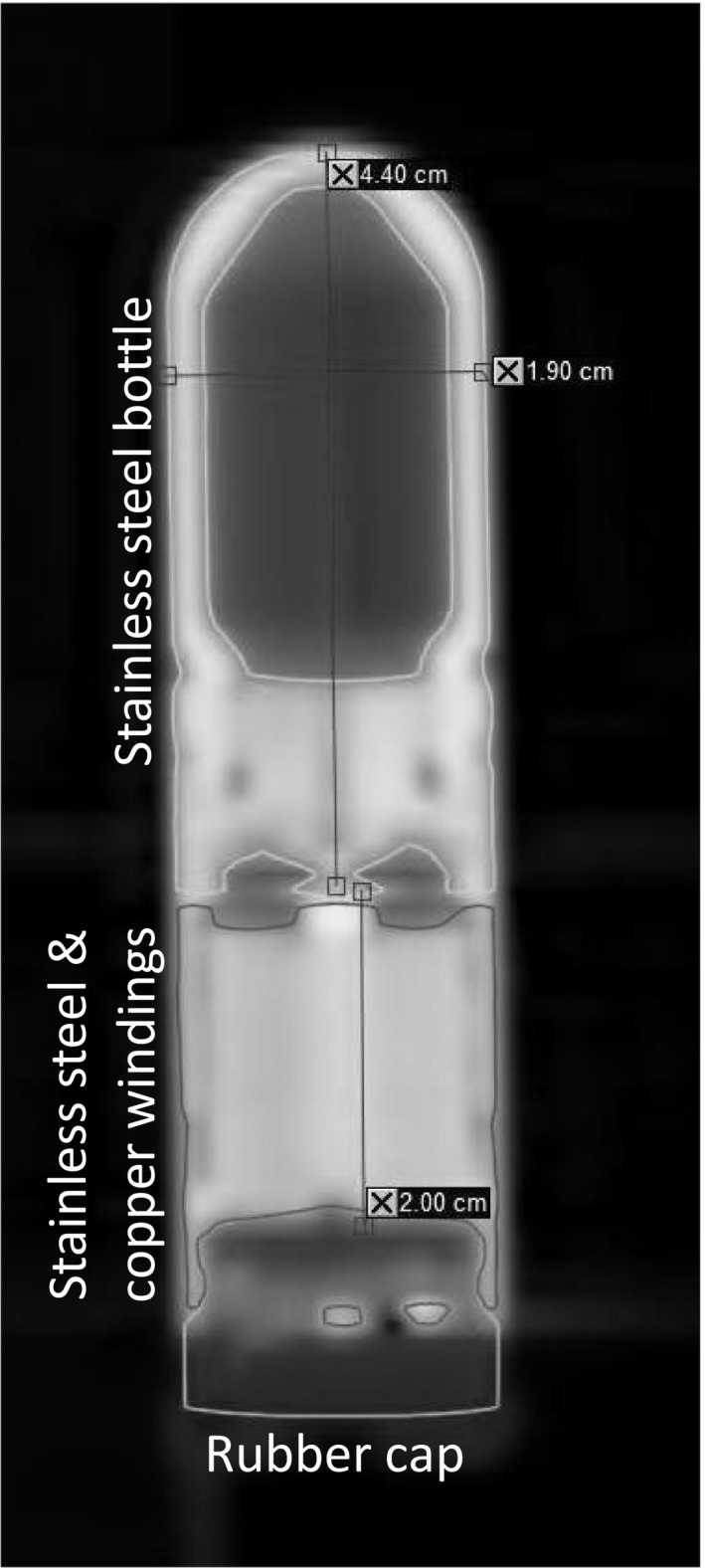
Coronal CT slice of the 400–600 cc AeroForm CO_2_ reservoir using optimal geometry and acquisition technique. Individual components are defined and verified with manufacturer specifications.

**Figure 3:**
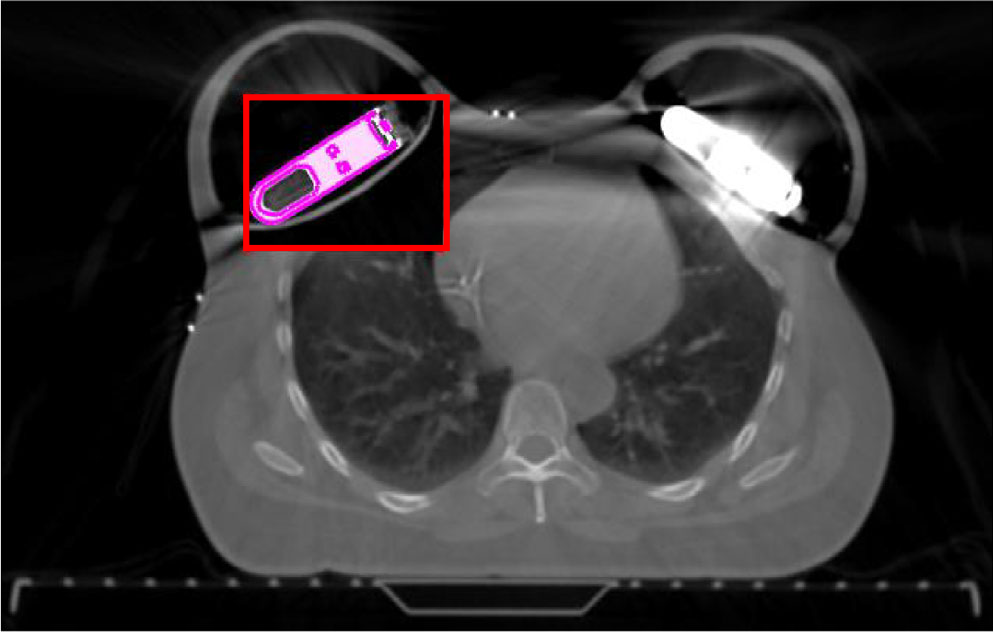
Bilateral AeroForm implants cause significant artifacts in the patient CT. Reservoir components are defined by registering the patient CT with to the treatment planning system model (shown in the “moving window”).

TPS models of both CO_2_ reservoir sizes were created in Eclipse (platform 13.5). The CT scans were imported into the TPS and individual components were identified and contoured using automatic thresholding techniques. The dimensions of the component structures were verified against manufacturer‐provided specifications. Figure. 2 details the individual components on a coronal CT slice. Using Eclipse, the TPS models were saved as phantom image and structure sets. The appropriate model could then be rigidly registered with any clinical patient planning CT series or experimental setup containing a TE, as shown in Fig. 3. This is particularly useful in clinical cases where artifacts are exacerbated by patient anatomy, expander orientation, or multiple expanders, where the expanders are difficult to define. Patient planning CTs were acquired using a typical breast protocol: 60 cm FOV with a 3 mm slice thickness using 120 kVp and 50 mAs. A 16‐bit reconstruction was generated both with and without MAR reconstruction.

### 2.2 Validation of the AeroForm TPS model

**Figure 4:**
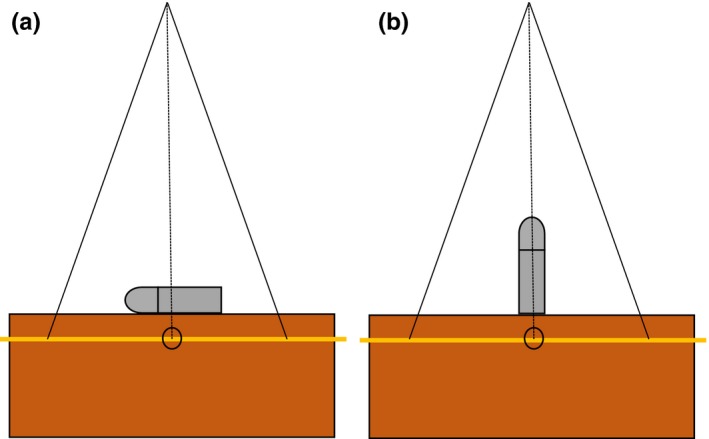
(a) Horizontal and (b) vertical orientations of the AeroForm TE for dose measurement. Gafchromic film is sandwiched between a 1 cm slab of solid water and 5 cm of solid water backscatter.

Several simple geometries were used to validate the TE models. Each expander was oriented vertically and horizontally on slabs of solid water, as shown in Fig. 4. A CT scan of each geometry was acquired and imported into Eclipse TPS. Individual components of each expander were defined by rigidly registering the acquired CT scan to the appropriate TPS model, as described previously. The solid water and air surrounding the expanders were also contoured, so appropriate densities could be assigned to correct for streaking artifacts. A single open field plan was created to deliver an AP, 20 cm^2^ × 20 cm^2^, 6 MV beam to each geometry. Dose was calculated using a 1 mm grid size for each expander size and geometry, with both AAA and AXB dose calculation algorithms (version 13.5.35).

The planned AP 20 cm^2 ^× 20 cm^2^ 6 MV beam was delivered to each set‐up using a Varian TrueBeam® linear accelerator (Varian Medical Systems, Palo Alto, CA, USA). For each geometry, a Gafchromic EBT3 film (Ashland Specialty Ingredients GP, Bridgewater, NJ, USA) was placed between solid water slabs at a depth of 1 cm (as shown in Fig. 4). The film was scanned using a Vidar Dosimetry Pro Advantage film digitizer (Vidar Systems Corporation, Herndon, VA, USA) according to departmental protocol.^23^ A calibration curve specific to the film batch and energy was created using the same protocol. The dose plane measured with film was compared to the dose plane calculated in Eclipse using Matlab (Mathworks, Natick, MA, USA) based in‐house software. Gamma index maps, and line profiles were generated to evaluate the accuracy of the two dose calculation methods for each set‐up. Gamma analysis was performed to quantify dose calculation accuracy. Gamma parameters of 3% dose difference and 3 mm distance to agreement were used for all analyses.

In the planning CT, density overrides were applied to the air and solid water to correct for artifacts. Initially, CT values of the various components of the CO_2_ reservoir were assigned based on manufacturer specifications. In AAA, initial CT values were assigned to achieve a physical density as near as possible to the vendor‐stated physical density. In AXB, initial CT values were assigned based on the Eclipse default material values for stainless steel. Upon analysis, the densities of the various AeroForm components were adjusted to best fit measured data. Re‐assignment of CT value, dose calculation, and comparison to measured data was then repeated iteratively to identify the optimal density assignments that resulted in the best agreement between measured and calculated data for both AAA and AXB.

### 2.3 Evaluation of the AeroForm TPS model in clinical cases

Patient data were analyzed as part of an IRB‐approved retrospective study. Four postmastectomy patients with AeroForm TEs were simulated. Patients 1, 2, and 4 had bilateral 600 cc AeroForm implants. Patient 3 had a single 800 cc AeroForm implant. All three patients were simulated on a Philips Brilliance Big Bore CT scanner, using helical CT scans, with 3‐mm slice thickness, 60 cm FOV, 120 kVp and 400 mAs and with a 16‐bit reconstruction. All CT scans were reconstructed both with and without MAR for evaluation. The non‐MAR scan was used as the primary planning CT, due to reconstruction artifacts (see section 3.A).

The components of the AeroForm device were defined by registering the appropriate TPS model to the planning CT. The expander balloon was also contoured: this structure includes the air cavity inside the AeroForm’s silicon shell, and excludes the metal CO_2_ reservoir. Normal critical structures were contoured and reviewed by the physician according to departmental protocol. The target breast was contoured by the physician. A structure called “PTV_EVAL” was created for plan evaluation. PTV_EVAL consists of the target breast as defined by the physician, minus the entire AeroForm expander (the CO_2_ reservoir and the expander balloon). PTV_EVAL represents the clinically significant patient tissue inside the target breast that requires adequate dose coverage.

**Figure 5:**
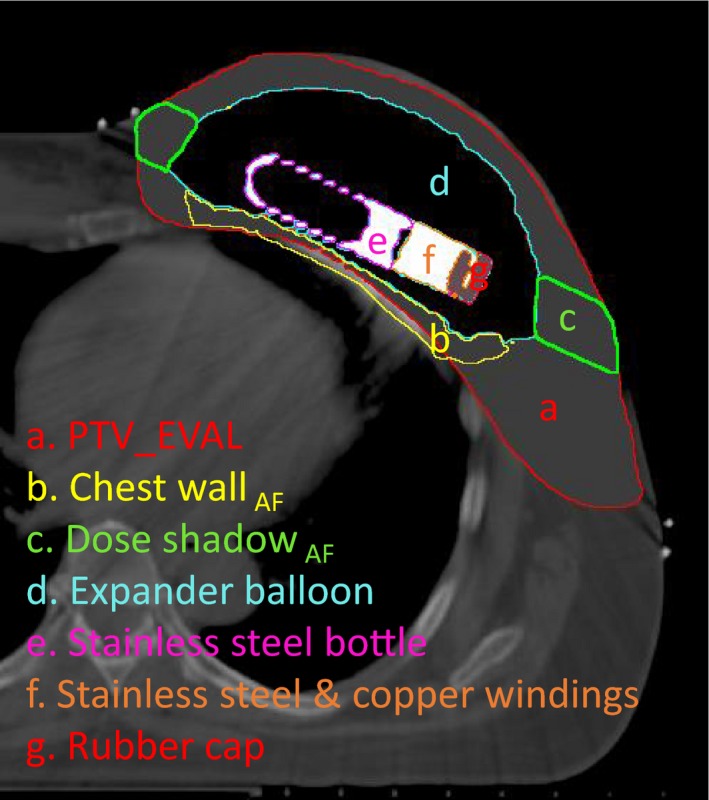
Axial CT of a patient breast with AeroForm TE, with structures created for density overrides and plan evaluation.

Two additional structures were defined for the purpose of evaluating areas of particular interest near the CO_2_ reservoir. “Chest wall _AF_” is defined as the tissue between the lung and expander balloon, and within 2 cm of the CO_2_ reservoir. “Dose shadow _AF_” is a cylindrical projection of the CO_2_ reservoir along its long axis in PTV_EVAL. Both structures are shown in Fig. 5.

The optimized CT values (Table 2) were assigned to all CO_2_ reservoir components. Additionally, density overrides were applied to the expander balloon and PTV_EVAL structures to correct for streaking artifacts. Density overrides were only necessary in the CT slices where the CO_2_ reservoir was visible.

**Figure 6:**
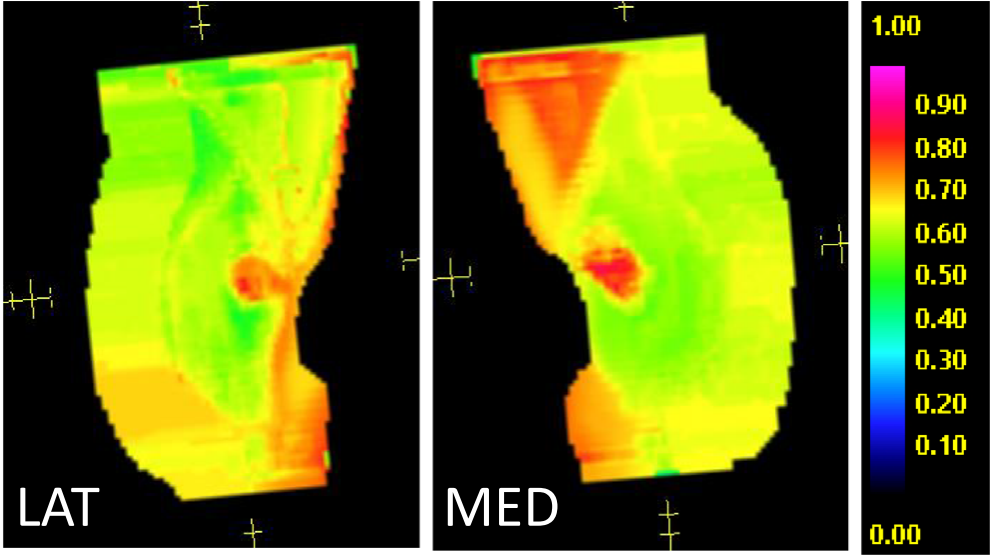
Fluence maps of tangent fields (Patient 2). To achieve adequate dose coverage, fluence is boosted in the chest wall and AeroForm dose shadow regions.

An experienced dosimetry team planned all four cases using 6 MV tangential geometries. All plans included the use of 0.5 cm of bolus over the entire breast every other day. Planning goals for AeroForm structures are shown in Table 1. Dose distributions were optimized using electronic tissue compensation (ECOMP) technique. To achieve acceptable coverage, the dose shadow and chest wall regions were “boosted.” An example of the resulting fluence is shown in Fig. 6.

**Table 1:** Planning goals for AeroForm structures.

Structure	Goal	Acceptable
PTV_EVAL	D_min_ > 5%	D_min_ > 90%
PTV_EVAL	D_max_ < 108%	D_1cc_ < 110%
Reservoir	D_max_ < 65Gy	D_max_ < 75Gy

Initial planning employed AAA dose calculation algorithm. After a satisfactory plan was achieved and reviewed by a physician, each plan was re‐calculated using AXB dose calculation algorithm. Beam parameters were unchanged. Patients 1, 3, and 4 were planned to a total dose of 50 Gy, and patient 2 was planned to a total dose of 50.4 Gy.

Dose distributions calculated by AAA and AXB were compared by evaluating dose volume histograms (DVHs) of the structures of interest defined above. For each of the patient, the mean (D_mean_) and maximum (D_max_) dose of each structure was tabulated for both the AAA‐ and AXB‐calculated plan. The differences between values calculated by the two algorithms are reported.

## 3 RESULTS & DISCUSSION

### 3.1 Imaging and modeling the AeroForm TE

Using optimal geometry and imaging technique, the AeroForm CO_2_ reservoir alone can be exquisitely imaged without a MAR reconstruction. The CT technique described previously was used to create the TPS models of the AeroForm device in Eclipse TPS (Fig. 2). Measured dimensions of the stainless steel CO_2_ bottle and the stainless steel and copper windings (SSCW) agreed within 1 mm of vendor provided specifications. Additionally, the TPS model included the rubber cap, for which the vendor did not provide specifications.

Unfortunately, such a CT technique is not practical for a patient breast simulation. First, the AeroForm TE is surgically placed with the long axis parallel to the axial plane. It is not possible to orient the patient on the CT to achieve the optimal imaging of the reservoir. Furthermore, many patients have bilateral TEs, increasing the streaking artifact effect on the axial slices where both reservoirs are visible. Finally, a small FOV, 1‐mm slice thickness CT protocol is not suitable for a breast CT acquisition.

**Figure 7:**
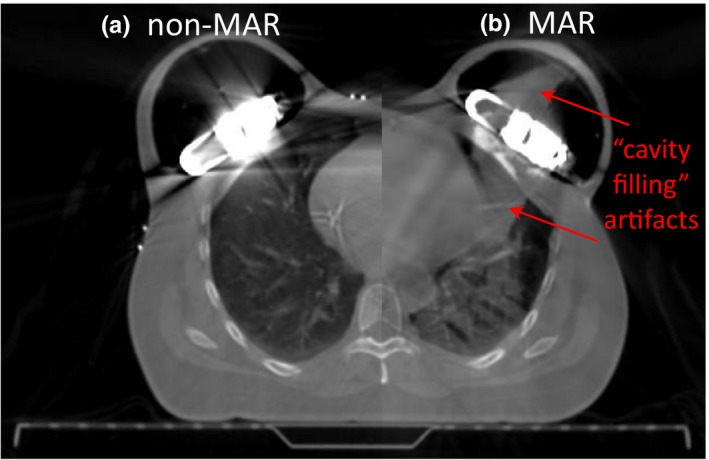
CT of a patient with bilateral AeroForm implants, split window between (a) non‐MAR and (b) MAR CT reconstructions. MAR causes “cavity filling” artifacts in the expander balloon and lungs.

Both reconstructions were made available during contouring, but the non‐MAR reconstruction was used as the primary planning CT. Planners observed that the MAR reconstruction offered better visualization of the CO_2_ reservoir and the external contour. However, near tissue‐air cavity interfaces such as the chest wall‐lung or PTV_EVAL‐expander balloon boundaries, the MAR reconstruction sometimes generated high‐density “cavity filling” artifacts, as shown in Fig. 7. Therefore, the non‐MAR reconstruction was more useful in defining the lungs and the expander balloon. Due to the severe streaking artifacts (particularly where patients had bilateral expanders), lung and external structures created using the auto‐contouring tools required manual editing on slices where the reservoir was visible.

### 3.2 Validation of the AeroForm TPS model

**Figure 8:**
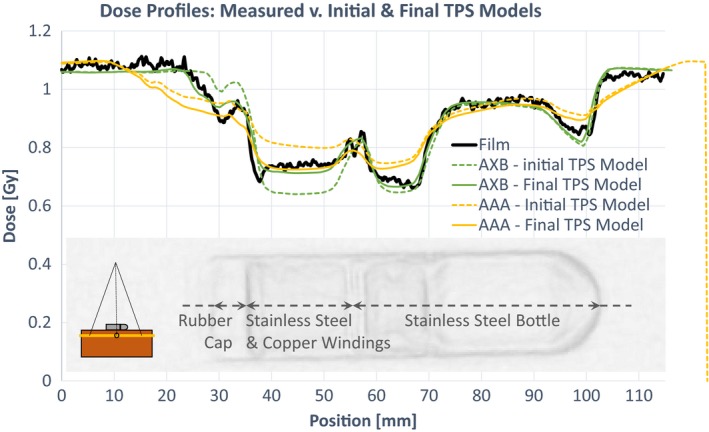
Measured and calculated dose profiles before and after relative electron density assignment optimization in the treatment planning system model. Schematic insert indicates geometry, reservoir overlay illustrates AeroForm component locations.

Transmission through the AeroForm CO_2_ reservoir was best modelled using the RED assignments shown in Table 2. Figure 8 compares the measured and calculated dose profiles along the long axis of the AeroForm for the horizontal geometry [Fig. 4(a)]. Calculated dose profiles are shown before and after the RED assignments were optimized, for both AAA and AXB. In AAA, the initial model (which assigned densities based on manufacturer specified physical densities) overestimated dose transmitted through both the stainless steel bottle (SSB) and the SSCW. After the RED of the SSCW was adjusted to 6.1 (Table 2), the measured and calculated transmission through the SSCW agreed within 3%. The RED of the SSB was assigned the maximum allowable value (6.58). However, even at the maximum value, AAA overestimated the transmission through the solid parts of the SSB. AAA did not accurately model the dose at and near boundaries of individual components. Where the film showed steep gradients between components, AAA calculated more rounded shoulders.

**Table 2:** Manufacturer specified physical densities and experimentally optimized RED values for various components of AeroForm TE.

Reservoir structure	*ρ* (g/cc)	RED AAA	RED AXB
Stainless steel CO_2_ bottle	8.1	6.6 (max)	6.1
Stainless steel & copper windings	5.5	6.1	5.1
Rubber cap	–	–	1.1

AAA, anisotropic analytical algorithm; AXB, acuros external beam; RED, relative electron density.

In AXB, the initial model (which assigned RED based on the Eclipse material density default value for stainless steel) underestimated transmission through the SSCW, and slightly underestimated transmission through the SSB. The density assignments were adjusted such that calculated and measured transmission through both materials agreed within 3%. The original TPS model did not include the rubber cap, as there were no vendor specifications for this component. However, exclusion of the cap caused a discrepancy between the calculated and measured dose in the affected region. Therefore, the cap was added to the TPS model. The cap dimensions were defined using the CT acquisition described in section 2.A, and the assigned RED was optimized in the same manner as the other components. The rubber cap was not included in the AAA model, because it exacerbated the dose shoulder effect evident at component boundaries in the AAA calculated dose.

**Figure 9:**
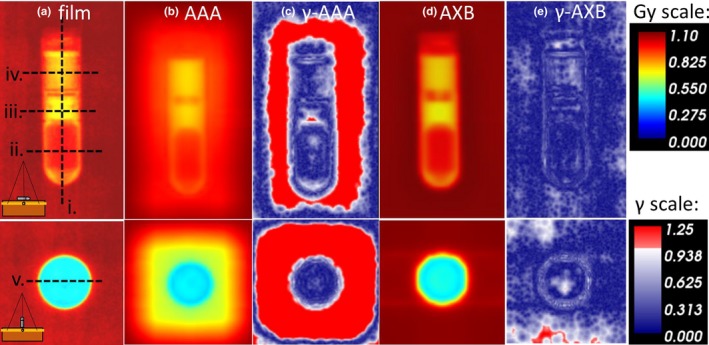
(a) Dose measured with film (b) Dose calculated with anisotropic analytical algorithm (AAA) (c) Film v. AAA Gamma analysis (d) Dose calculated with Acuros External Beam (AXB) (e) Film v. AXB Gamma analysis. Schematic insert indicates geometry. Dashed lines indicate the dose profiles shown in Fig 10.

Figure 9 shows Gamma analysis results from the film measurement. Using optimized RED values, Gamma pass rates in the region where the reservoir directly attenuated the beam were greater than 99% for both AAA and AXB. However, in the region directly adjacent to the reservoir (between 0 and 1 cm from the device edge), AAA consistently underestimated dose and nearly all points within 1 cm failed the gamma criteria. For AXB, Gamma pass rates in this region were greater than 98%.

**Figure 10:**
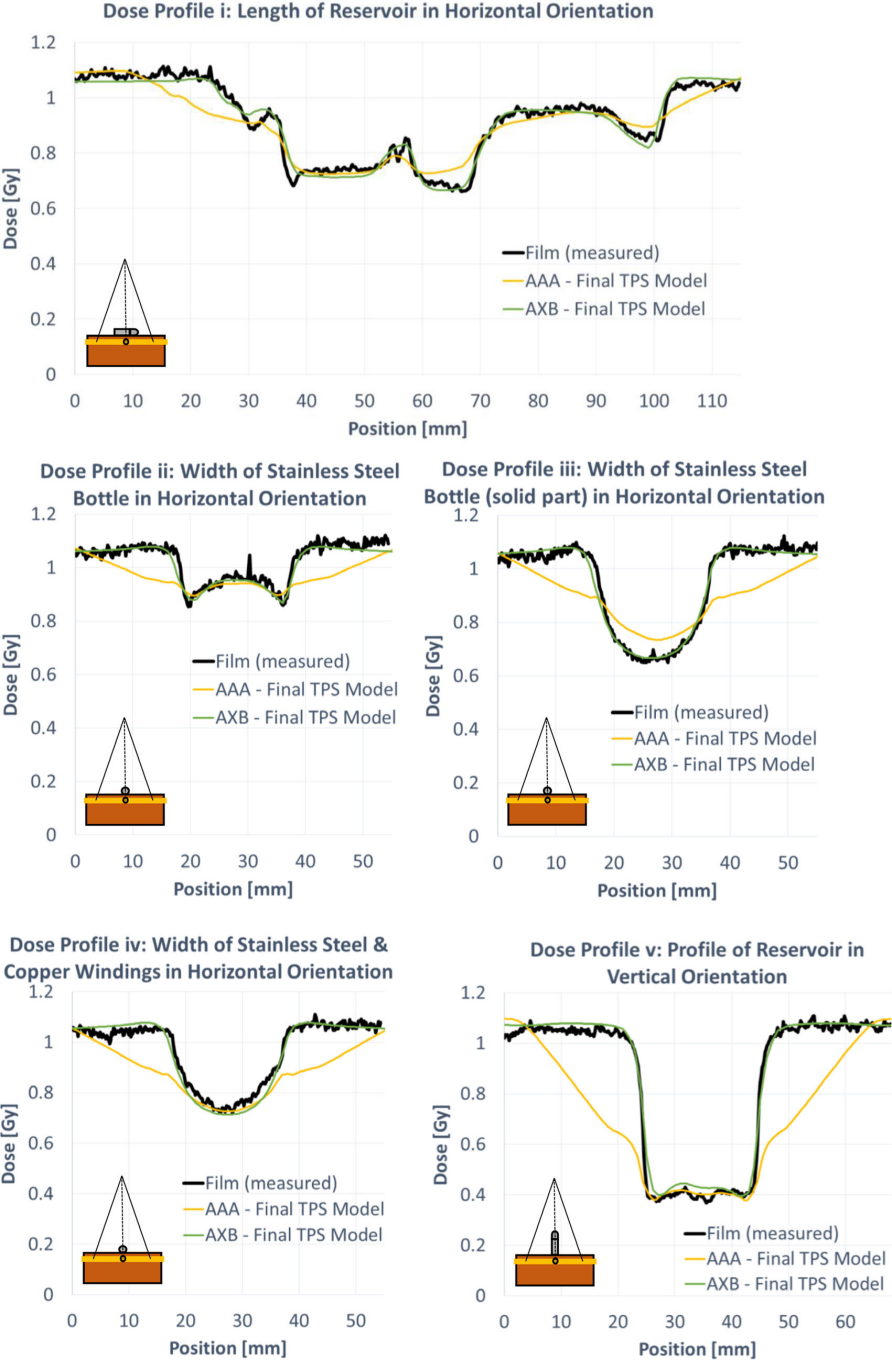
Measured (film) and calculated dose profiles comparing the AAA and AXB TPS model in various orientations, as shown by the schematic inserts. The dashed lines in Fig. 8 indicate the locations of each profile: (i) length of horizontal reservoir, width of horizontal reservoir through the (ii) stainless steel bottle (iii)solid stainless steel (iv) copper windings, and (v) width of vertical reservoir in Fig. 8.

Figure 10 shows various profiles of the film and AAA‐ and AXB‐calculated dose planes. The dashed lines in Fig. 9(a) indicate the location of each profile. Using the optimized RED overrides, both calculation models adequately predict transmission directly through the reservoir. However, AAA underestimates dose in the region immediately adjacent to the reservoir. Additionally, the maximum RED value allowed by the AAA dose calculation algorithm is 6.5845. Even using the maximum RED value, transmission through the solid part of the SSB is slightly overestimated by AAA.

### 3.3 Evaluation of the AeroForm TPS model in clinical cases

**Figure 11:**
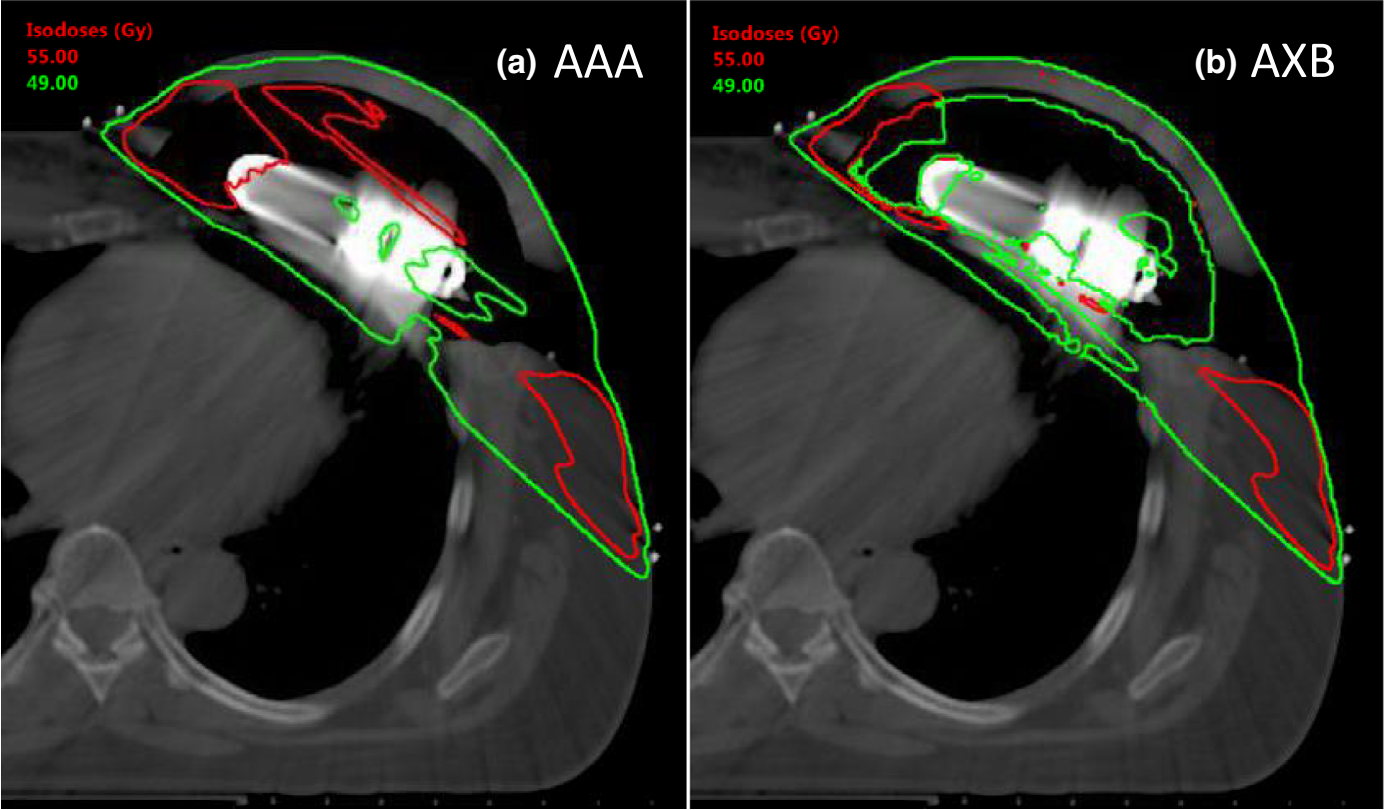
Dose distribution for Patient 3 calculated with (a) AAA and (b) AXB. Compared to AXB, AAAoverestimates dose in the expander balloon and reservoir. The dose distribution in PTV_EVAL is mostaffected in the chest wall region adjacent to the reservoir.

**Figure 12:**
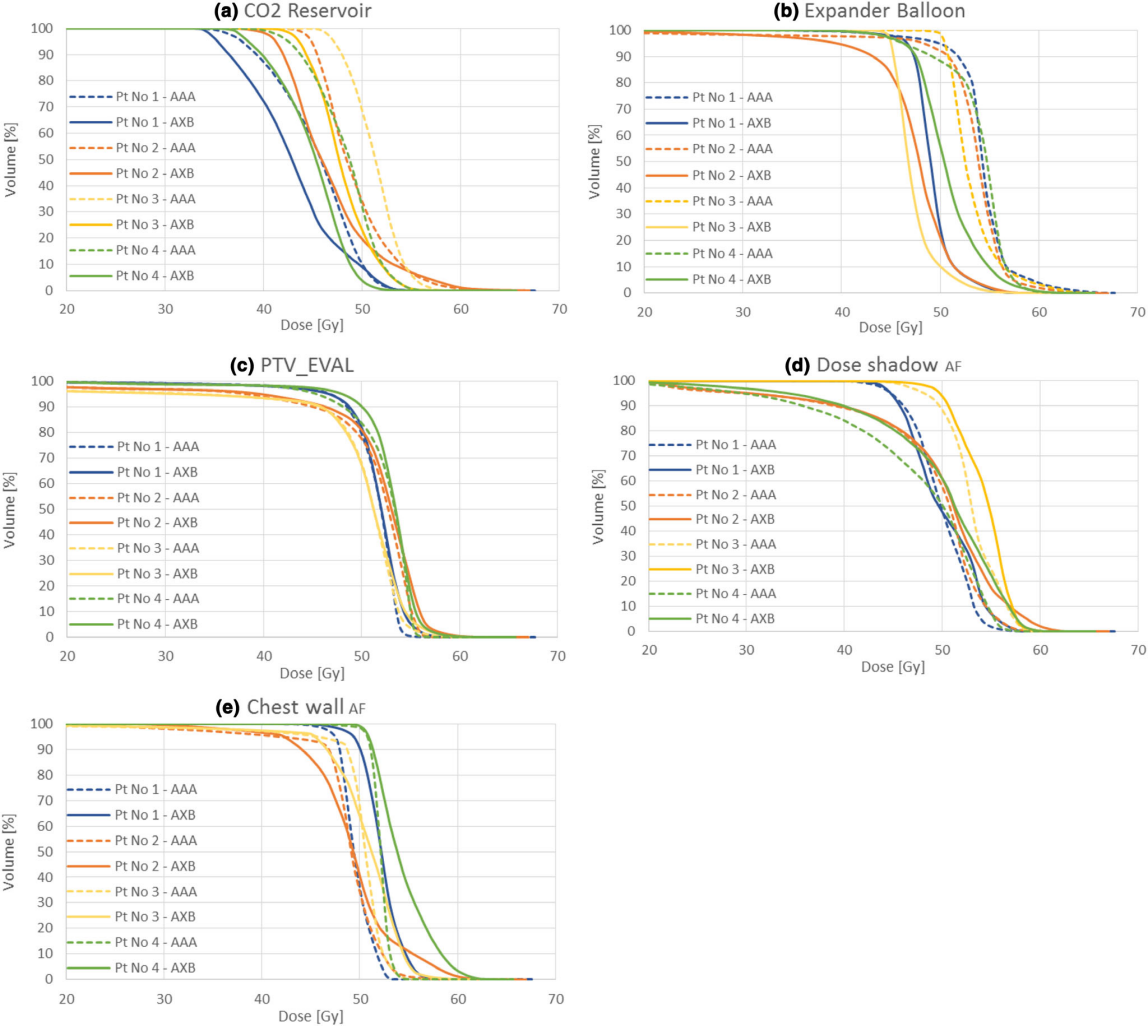
DVHs for structures of interest (a) CO2 reservoir (b) expander balloon (c) PTV_EVAL (d) doseshadow AF (e) chest wall AF Four patient plans were calculated using AAA and AXB..

An example planned dose distribution for Patient 3 is shown in Fig. 11, as calculated by (a) AAA and (b) AXB, (both using the optimized AeroForm TPS model). The plan was originally calculated using AAA, and optimized using ECOMP technique. When the plan was recalculated using AXB, the dose in the CO_2_ reservoir and expander balloon decreased. However, this change may not be clinically relevant, as these structures do not include patient tissue. The locations and magnitudes of hot spots in the PTV_EVAL do not appear to be greatly altered. A change in dose distribution is evident at the chest wall region adjacent to the reservoir. Similar differences and similarities between AAA and AXB were observed for all four patients. Figure 12 shows DVH plots of various structures of interest for all patients. The mean and maximum doses of each structure are tabulated in Tables 3 and 4, respectively. The percent difference between the AAA and AXB calculated doses are also reported in Tables 3 and 4.

**Table 3:** Mean dose for various regions of interest, calculated with AAA and AXB.

Mean dose	Patient No. 1			Patient No. 2			Patient No. 3			Patient No. 4		
	AAA (Gy)	AXB (Gy)	Δ (%)	AAA (Gy)	AXB (Gy)	Δ (%)	AAA (Gy)	AXB (Gy)	Δ (%)	AAA (Gy)	AXB (Gy)	Δ (%)
CO_2_ Reservoir	45.2	42.9	5.4%	49.2	46.8	5.0%	51.2	48.0	6.9%	48.3	44.8	7.7%
Expander balloon	54.3	49.2	10.4%	53.2	47.3	12.5%	53.2	47.3	12.6%	53.8	50.7	6.2%
PTV_EVAL	51.1	51.4	−0.6%	50.6	51.3	−1.3%	49.1	49.2	−0.3%	52.1	52.8	−1.3%
Chest wall AF	49.6	52.2	−5.0%	48.7	49.2	−1.1%	49.9	50.4	−1.1%	52.1	54.4	−4.2%
Dose shadow AF	49.8	50.1	−0.5%	48.3	49.3	−2.0%	53.0	54.2	−2.2%	47.2	49.6	−4.8%

AAA, Anisotropic Analytical Algorithm; AXB, Acuros External Beam.

**Table 4:** Maximum dose for various regions of interest, calculated with AAA and AXB.

Maximum dose	Patient No. 1			Patient No. 2			Patient No. 3			Patient No. 4		
	AAA (Gy)	AXB (Gy)	Δ (%)	AAA (Gy)	AXB (Gy)	Δ (%)	AAA (Gy)	AXB (Gy)	Δ (%)	AAA (Gy)	AXB (Gy)	Δ (%)
CO_2_ Reservoir	60.7	55.7	8.9%	63.9	67.1	−4.7%	60.4	60.4	−0.1%	57.8	55.4	4.2%
Expander balloon	67.7	58.7	15.4%	65.9	64.3	2.6%	65.6	61.6	6.4%	65.8	65.7	0.2%
PTV_EVAL	62.1	59.4	4.5%	62.3	63.5	−1.9%	62.3	61.9	0.6%	62.7	65.4	−4.1%
Chest wall AF	53.5	58.0	−7.8%	62.3	63.5	−1.9%	60.1	61.4	−2.1%	58.8	65.3	−10.0%
Dose shadow AF	59.6	58.4	2.0%	61.3	62.9	−2.5%	60.3	60.7	−0.6%	61.9	61.6	0.5%

AAA, Anisotropic Analytical Algorithm; AXB, Acuros External Beam.

The DVH plots show that, compared to AXB, AAA consistently overestimates dose in the CO_2_ reservoir and in the expander balloon. D_mean_ of the CO_2_ reservoir is between 5% and 8% higher when calculated by AAA. D_mean_ of the expander balloon is 6 to 13% higher when calculated by AAA. The difference in D_max_ is highly variable.

The changes in dose distribution in patient tissue are more complex. In evaluating the entire PTV_EVAL volume, differences between AAA and AXB appear to be minimal; changes in mean dose to PTV_EVAL are less than 1.5% for all patients. Differences in specific subregions of PTV_EVAL appear to be somewhat patient‐specific. AAA underestimated D_mean_ to the chest wall near the CO_2_ reservoir (chest wall _AF_) by 1.0% to 5.0%. The shape of the DVH shoulder and tail was sharper in AAA than in AXB for patients 2, 3, and 4, whereas the DVH for patient 1 appeared to be uniformly shifted. D_mean_ in the dose shadow region was between 0% and 5% lower when calculated using AAA compared to AXB. Changes in DVH shape did not follow a distinct pattern. This may indicate that dose distribution differences in the dose shadow and chest wall regions are anatomy‐ and plan‐specific and cannot be easily generalized. Changes in D_max_ were highly variable. This is partially explained by the fact that plan optimization using AAA aimed specifically to control hot spots, whereas the re‐calculation with AXB did not include any re‐optimization.

Differences in D_max_ and in the shoulder and tail regions of clinically significant structure DVHs may have a significant impact on plan outcome. If the clinician decides to use a simple planning technique such as wedged tangents, the AeroForm TE will decrease plan quality in a relatively predictable way: compared to an intact breast, dose will decrease in the dose shadow region and in the chest wall. The clinician must decide whether the suboptimal dose distribution will still accomplish clinical goals. Improved planned target coverage and hot‐spot control might be achieved with advanced plan optimization techniques, such as ECOMP, field‐in‐field, or another form of inverse optimization. These techniques modulate fluence to limit dose to hot spots, and increase dose to cold spots. However, with these techniques, the precise location and value of hot and cold spots (thus the shape of the target DVH in the shoulder and tail region) will inform the planned beam modulation. As discussed previously, D_max_ differences between AAA and AXB are variable, and therefore advanced plan optimization using AAA versus AXB could result in a significantly different plans. If, for example, AAA underestimates dose to the chest wall, an optimized plan in AAA would increase fluence to this region in order to achieve good coverage. But calculated using AXB, the same plan might have an unacceptable hot spot in the chest wall. If advanced planning techniques will be used, it is essential that accurate CT data and dose calculation methods are available.

## 4 CONCLUSION

The AeroForm TE poses significant challenges in a radiation therapy setting. A clinician must decide whether to treat patients with the implant, what planning technique to use, and how to evaluate plan quality. To make these decisions, it is essential they have accurate CT and dose data, and understand the limitations of their TPS.

This study describes a technique to model the AeroForm TE in a commercially available TPS. The physical dimensions of the TPS model agreed with vendor specified values. The TPS model therefore gives an accurate picture of the TE structure within the patient anatomy. Near AeroForm TEs, patient CT images suffer from significant streaking artifacts, and a MAR reconstruction may not be an adequate solution. By facilitating accurate delineation of relevant anatomy, the TPS model overcomes some challenges of contouring and plan evaluation on a CT image with poor image quality due to artifact.

The RED assignments for the TE components were optimized experimentally to maximize dose calculation accuracy. This resulted in good agreement between measured and calculated dose for AXB dose calculation algorithm. Agreement between measured and calculated dose for AAA was acceptable in regions of direct transmission, but AAA underestimated dose in the regions adjacent to the reservoir. This result demonstrates the limits of a convolution‐based dose calculation algorithm. For clinics with multiple algorithms available, selecting an algorithm that accounts for effects of electron transport (such as the AXB algorithm used in this study) will maximize dose accuracy in the vicinity of the TE. For both algorithms, an optimized TPS model of the TE will improve dose calculation accuracy.

The increased accuracy of the optimized TPS model, used in conjunction with AXB algorithm, has a clinical benefit when applied to patient data. The AeroForm TE presents unique challenges in achieving adequate dose coverage of the target, particularly in the dose shadow and chest wall regions. Advanced treatment planning techniques can be employed to achieve coverage in these areas. However, techniques such as ECOMP, field‐in‐field, or inverse optimization, rely on accurate dose calculations. This study compared dose calculated by AAA and AXB for ECOMP plans in clinical patient data using the AeroForm TPS model. Dose differences between the two algorithms existed in clinically relevant regions of patient anatomy, such as the dose shadow, and the chest wall. Calculated dose in these areas informs the optimization of the treatment plan, and thus directly affects the design of treatment beams. Beam optimization, and therefore target coverage, will be improved using the optimized TPS model of AeroForm in conjunction with a deterministic dose calculation algorithm such as AXB.

## CONFLICTS OF INTEREST

The authors declare no conflict of interest.
